# 1-(Bromo­mercurio)ferrocene

**DOI:** 10.1107/S1600536813032923

**Published:** 2013-12-11

**Authors:** Jens M. Breunig, Hans-Wolfram Lerner, Jan W. Bats

**Affiliations:** a Institut für Anorganische und Analytische Chemie, Universität Frankfurt, Max-von-Laue-Strasse 7, D-60438 Frankfurt am Main, Germany; b Institut für Organische Chemie und Chemische Biologie, Universität Frankfurt, Max-von-Laue-Strasse 7, D-60438 Frankfurt am Main, Germany

## Abstract

The asymmetric unit of the title compound, [Fe(C_5_H_5_)(C_5_H_4_BrHg)], contains two independent mol­ecules, *A* and *B*, in which the Hg—C bond lengths are 2.045 (6) and 2.046 (6) Å, the Hg—Br bond lengths are 2.4511 (9) and 2.4562 (7) Å, and the C—Hg—Br angles are 176.42 (17) and 177.32 (17)°. The two cyclo­penta­dienyl rings of mol­ecule *A* are eclipsed, while those of mol­ecule *B* are almost staggered. The HgBr groups are connected by inter­molecular Hg⋯Br contacts of 3.3142 (9)–3.4895 (11) Å, forming layers parallel to (001). These layers contain both four-membered (HgBr)_2_ and eight-membered (HgBr)_4_ rings. Ferrocene–ferrocene C—H⋯π contacts connect the mol­ecular layers along the *c*-axis direction.

## Related literature   

For synthetic background, see: Fish & Rosenblum (1965 ▶); Guillaneux & Kagan (1995 ▶). For chemical background, see: Lerner (2005 ▶). For related structures, see: Meyer-Wegner *et al.* (2012 ▶); Hayashi *et al.* (2011 ▶); Franz *et al.* (2011 ▶); Wiberg *et al.* (1997 ▶, 2001 ▶); Margraf *et al.* (2004 ▶); Sünkel & Kiessling (2001 ▶); Romanov *et al.* (2007 ▶); Singh *et al.* (2005 ▶). For van der Waals radii, see: Bondi (1964 ▶).
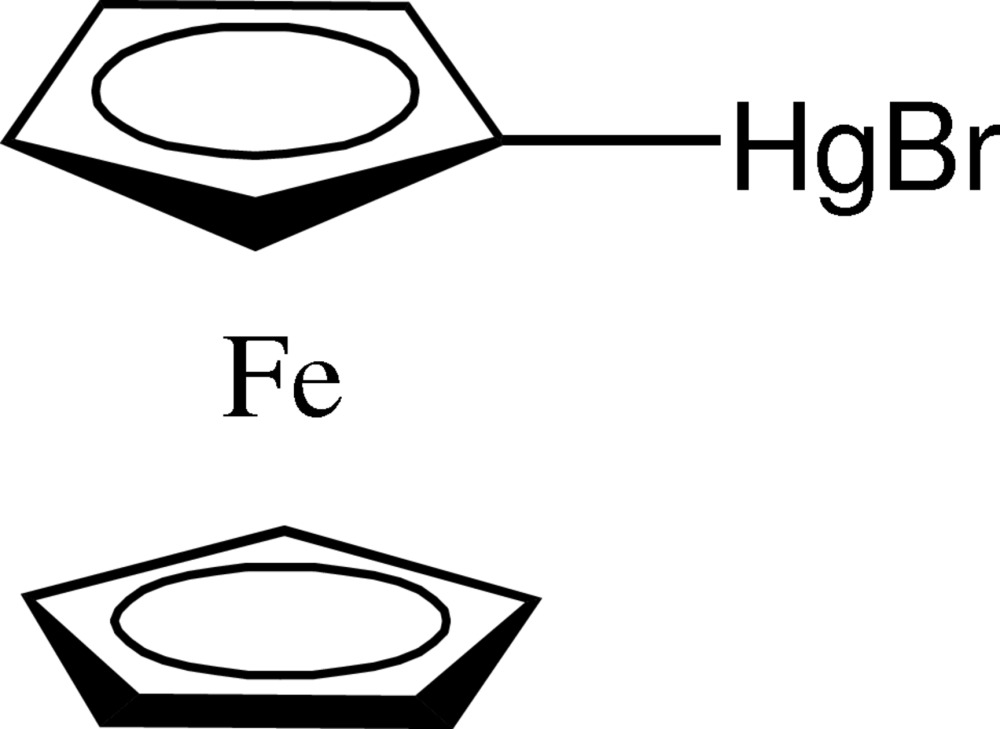


## Experimental   

### 

#### Crystal data  

[FeHgBr(C_5_H_5_)(C_5_H_4_)]
*M* *_r_* = 465.52
Triclinic, 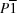

*a* = 7.6484 (14) Å
*b* = 9.5715 (17) Å
*c* = 14.394 (3) Å
α = 75.120 (12)°
β = 87.548 (12)°
γ = 83.298 (12)°
*V* = 1011.3 (3) Å^3^
*Z* = 4
Mo *K*α radiation
μ = 20.49 mm^−1^
*T* = 166 K
0.65 × 0.28 × 0.05 mm

#### Data collection  

Siemens SMART 1K CCD diffractometer
Absorption correction: numerical (*SHELXTL*; Sheldrick, 2008 ▶) *T*_min_ = 0.010, *T*_max_ = 0.361
16880 measured reflections
5759 independent reflections
4942 reflections with *I* > 2σ(*I*)
*R*_int_ = 0.081

#### Refinement  

*R*[*F* ^2^ > 2σ(*F* ^2^)] = 0.035
*wR*(*F* ^2^) = 0.091
*S* = 1.10
5759 reflections
236 parameters
H-atom parameters constrained
Δρ_max_ = 2.78 e Å^−3^
Δρ_min_ = −1.93 e Å^−3^

### 

Data collection: *SMART* (Siemens, 1995 ▶); cell refinement: *SMART*; data reduction: *SAINT* (Siemens, 1995 ▶); program(s) used to solve structure: *SHELXS97* (Sheldrick, 2008 ▶); program(s) used to refine structure: *SHELXL97* (Sheldrick, 2008 ▶); molecular graphics: *SHELXTL* (Sheldrick, 2008 ▶); software used to prepare material for publication: *SHELXL97*.

## Supplementary Material

Crystal structure: contains datablock(s) default, I. DOI: 10.1107/S1600536813032923/nc2322sup1.cif

Structure factors: contains datablock(s) I. DOI: 10.1107/S1600536813032923/nc2322Isup2.hkl

Additional supporting information:  crystallographic information; 3D view; checkCIF report

## Figures and Tables

**Table 1:** Hydrogen-bond geometry (Å, °) *Cg*1 and *Cg*2 are the centroids of the C6–C10 and C16–C20 rings, respectively.

*D*—H⋯*A*	*D*—H	H⋯*A*	*D*⋯*A*	*D*—H⋯*A*
C3—H3*A*⋯*Cg*2^iv^	0.95	2.91	3.794 (8)	156
C19—H19*A*⋯*Cg*1^iii^	0.95	2.78	3.618 (8)	148

Symmetry codes: (iii) 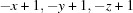; (iv) 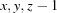.

**Fig. 1.**
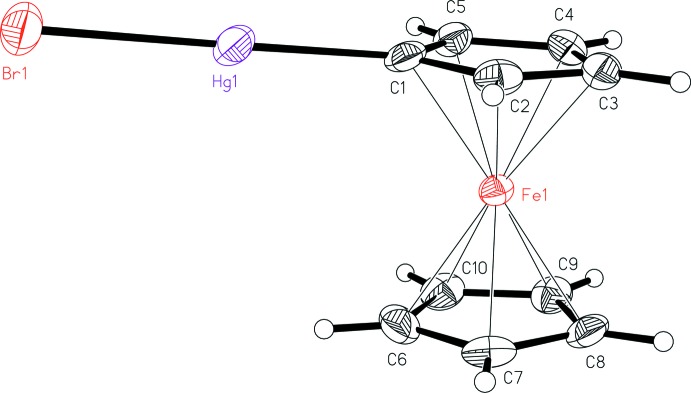
The structure of molecule A, showing the atom-labelling scheme. Displacement ellipsoids are drawn at the 50% probability level. H atoms are drawn as small spheres of an arbitrary radius.

**Fig. 2.**
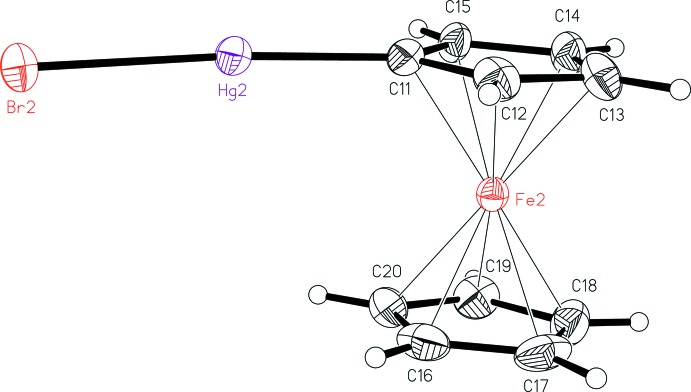
The structure of molecule B, showing the atom-labelling scheme. Displacement ellipsoids are drawn at the 50% probability level. H atoms are drawn as small spheres of an arbitrary radius.

**Fig. 3.**
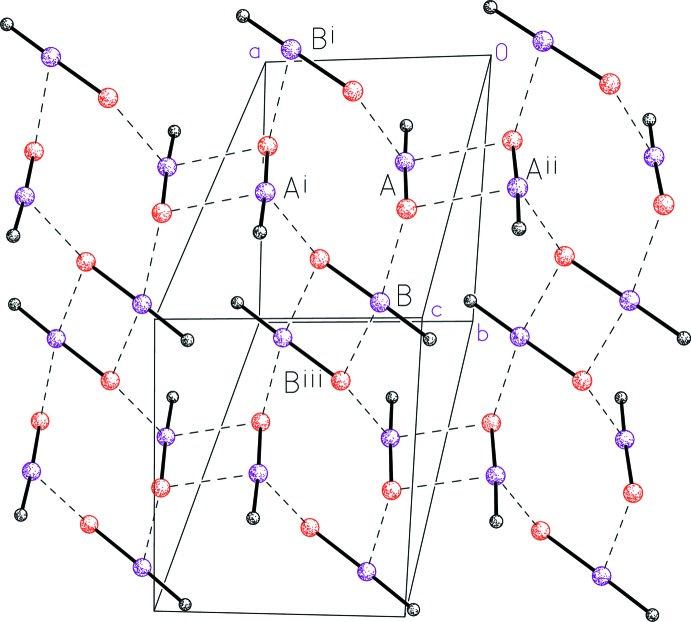
A layer of molecules connected by intermolecular Hg···Br contacts (broken lines). Arbitrary view direction near [0–11]. The ferrocene groups, except for the Hg-bound C atoms, have been omitted for clarity. Colour codes: C black, Hg purple, Br red. Symmetry codes: (i) -x + 1, -y, -z + 1, (ii) -x, -y, -z + 1, (iii) -x + 1, -y + 1, -z + 1. A and B represent molecules A and B.

**Fig. 4.**
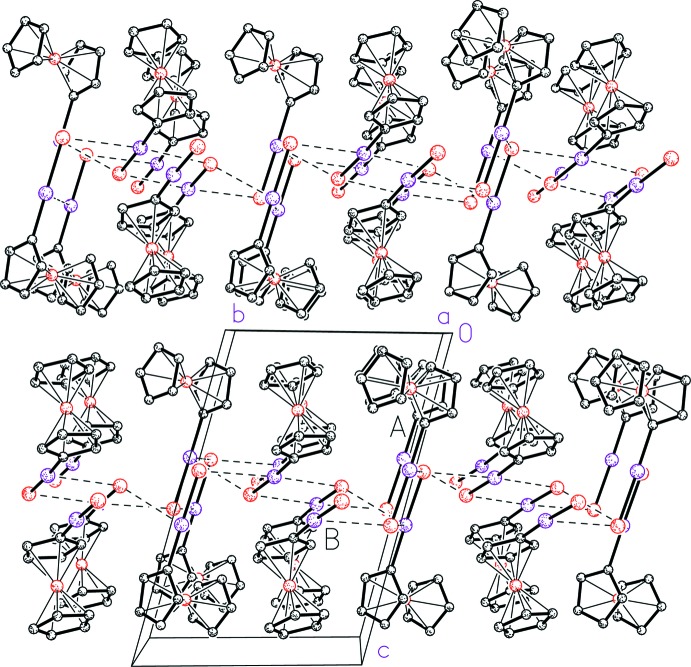
The crystal packing of the title compound viewed down [100]. H atoms have been omitted for clarity. Intermolecular Hg···Br contacts are shown as broken lines. A and B represent molecules A and B. Colour codes as for Fig. 3.

## supplementary crystallographic information

### 1. Comment

Our group has recently reported on the chemical and structural features of compounds containing the group 12 elements Zn, Cd and Hg (Meyer-Wegner *et al.*, 2012; Hayashi *et al.*, 2011; Franz *et al.*, 2011; Lerner, 2005; Wiberg *et al.*, 1997; Wiberg *et al.*, 2001; Margraf *et al.*, 2004). To extend our long-standing investigations in this field, we are currently studying the title compound 1-(bromomercurio)ferrocene. A protocol for the synthesis of 1-(chloromercurio)ferrocene has been reported by Fish & Rosenblum (1965). However, we were not able to prepare the title compound using that method. Therefore we decided to synthesize the compound *via* a new route by the reaction of 1-(tri-*n*-butylstannyl)ferrocene with HgBr_2_ (see experimental section). Here we report the crystal structure of the resulting compound.

The asymmetric unit of the title compound contains two independent molecules (*A*, Fig.1 and *B*, Fig. 2). The molecular conformations of the ferrocene groups are different: the two cyclopentadienyl rings of molecule *A* are eclipsed while those of molecule *B* are almost staggered. The HgBr group is slightly displaced from the cyclopentadienyl ring to which the Hg atom is attached [deviations from plane of five-membered ring: Hg1 0.096 (10) Å, Br1 0.263 (17) Å, Hg2 0.067 (11) Å, Br2 0.293 (19) Å]. This out-of-plane distortion is in opposite directions for molecules *A* and *B* and therefore may result mainly from intermolecular interactions·The angle between the planes of the two cyclopentadienyl rings is 3.1 (4)° for molecule *A* and 2.1 (5)° for molecule *B*.

For the corresponding 1-(chloromercurio)ferrocene two polymorphic crystal structures have been reported, a triclinic structure (Sünkel & Kießling, 2001) and a monoclinic structure (Romanov *et al.*, 2007). The title compound 1-(bromomercurio)ferrocene is isomorphous with the triclinic polymorph of 1-(chloromercurio)ferrocene.

Each HgBr group is connected by four long Hg···Br contacts to three neighboring HgBr groups as shown in Fig. 3. These additional Hg···Br contacts result in layers of molecules parallel to the (001) plane. Molecule *A* is connected by two Hg···Br contacts to a symmetry-related molecule *A* to form a four-membered (HgBr)_2_ ring. A similar four-membered ring is formed by two symmetry-related molecules *B*. Two molecules *A* and two molecules *B* are connected by four additional Hg···Br contacts to form eight-membered (HgBr)_4_ rings. No intermolecular Hg···Br contacts occur in the crystal structure of 1-(bromomercurio)-2,4,6-trimethylbenzene (Meyer-Wegner *et al.*, 2012). There an undistorted HgBr single bond length of 2.4307 (8) Å was found which is about 0.02 Å shorter than the values of 2.4511 (9) and 2.4562 (7) Å observed for molecules *A* and *B* of the title compound. In the crystal structure of 1-(bromomercurio)-4-methoxybenzene (Singh *et al.*, 2005) each HgBr bond is connected to the HgBr bonds of four neighboring molecules *via* intermolecular Hg···Br contacts of 3.4041 (7) to 3.5461 (7) Å and the HgBr single bond length is elongated to 2.4700 (7) Å. Although the long intermolecular Hg···Br contact distances are near the value of 3.40 Å, generally taken as the non-bonding distance between Hg and Br (Bondi, 1964), they do result in a small lengthening of the HgBr single bond.

The layers are connected along the *c*-axis direction by ferrocene···ferrocene contacts (Fig. 4). There is a weak intermolecular C_ferrocene_—H···π_ferrocene_ interaction between the C3—H3A bond and the cyclopentadienyl ring labelled C16—C20 from an adjacent layer (first entry in Table 1). An additional C_ferrocene_—H···π_ferrocene_ interaction connects the ferrocene groups within a single layer (second entry in Table 2).

### 2. Experimental

The starting material 1-(tri-*n*-butylstannyl)ferrocene was prepared as described by Guillaneux & Kagan (1995). The starting material (5.583 g, 11.75 mmol) was dissolved in CH_2_Cl_2_ (20 ml) and was added to HgBr_2_ (4.248 g, 11.79 mmol, 1 equivalent) in CH_2_Cl_2_ (80 ml). The reaction mixture was stirred for 30 minutes at room temperature and then filtered. All volatiles were removed in *vacuo* and the resulting solid residue was washed with *n*-hexane (250 ml). Drying to constant mass yielded the title compound 1-(bromomercurio)ferrocene (3.714 g, 7.978 mmol, 68%). The compound was recrystallized from CH_2_Cl_2_/*n*-pentane (1:1), resulting in brown blades. ^1^H and ^13^C{^1^H} NMR spectra were recorded at 298 K on a Bruker Avance 300 spectrometer using D_6_-benzene as solvent. Chemical shifts are reported in p.p.m. relative to Si(CH_3_)_4_ and were referenced to residual solvent signals. ^1^H (300.0 MHz): *δ*=3.48 (vtr, ^3^*J*_HH_=^4^*J*_HH_=1.7 Hz, 2H, Ar—H), 3.84 (s, 5H, C_5_H_5_), 4.05 (vtr, ^3^*J*_HH_=^4^*J*_HH_=1.7 Hz, 2H, Ar—H); ^13^C{^1^H} (100.6 MHz): *δ*=68.6 (C_5_H_5_), 69.7 (Ar—C), 72.7 (Ar—C), n.o. (*ipso*-C). Abbreviations: s=singlet, vtr=virtual triplet, n.o.=signal not observed. Elemental analysis (%) for C_10_H_9_BrFeHg: calculated C 25.80, H 1.95; found: C 25.52, H 1.96.

### 3. Refinement

H atoms were positioned geometrically and treated as riding: C—H=0.95 Å and *U*_iso_(H)=1.2*U*_eq_(C). The minimum and maximum residual peaks of -1.93 and +2.78 e.Å^-3^ in the difference Fourier synthesis are found at about 0.9 Å from the Hg atoms.

### Crystal data

**Table:**

[FeHgBr(C_5_H_5_)(C_5_H_4_)]	*Z* = 4
*M**_r_* = 465.52	*F*(000) = 840
Triclinic, *P*1	*D*_x_ = 3.057 Mg m^−^^3^
Hall symbol: -P 1	Mo *K*α radiation, λ = 0.71073 Å
*a* = 7.6484 (14) Å	Cell parameters from 153 reflections
*b* = 9.5715 (17) Å	θ = 3–23°
*c* = 14.394 (3) Å	µ = 20.49 mm^−^^1^
α = 75.120 (12)°	*T* = 166 K
β = 87.548 (12)°	Blade, brown
γ = 83.298 (12)°	0.65 × 0.28 × 0.05 mm
*V* = 1011.3 (3) Å^3^	

### Data collection

**Table:**

Siemens SMART 1K CCD diffractometer	5759 independent reflections
Radiation source: normal-focus sealed tube	4942 reflections with *I* > 2σ(*I*)
Graphite monochromator	*R*_int_ = 0.081
ω scans	θ_max_ = 30.0°, θ_min_ = 2.2°
Absorption correction: numerical (*SHELXTL*; Sheldrick, 2008)	*h* = −10→10
*T*_min_ = 0.010, *T*_max_ = 0.361	*k* = −13→13
16880 measured reflections	*l* = −20→20

### Refinement

**Table:**

Refinement on *F*^2^	Secondary atom site location: difference Fourier map
Least-squares matrix: full	Hydrogen site location: inferred from neighbouring sites
*R*[*F*^2^ > 2σ(*F*^2^)] = 0.035	H-atom parameters constrained
*wR*(*F*^2^) = 0.091	*w* = 1/[σ^2^(*F*_o_^2^) + (0.037*P*)^2^ + 4*P*] where *P* = (*F*_o_^2^ + 2*F*_c_^2^)/3
*S* = 1.10	(Δ/σ)_max_ = 0.001
5759 reflections	Δρ_max_ = 2.78 e Å^−^^3^
236 parameters	Δρ_min_ = −1.93 e Å^−^^3^
0 restraints	Extinction correction: *SHELXL97* (Sheldrick, 2008), Fc^*^=kFc[1+0.001xFc^2^λ^3^/sin(2θ)]^-1/4^
Primary atom site location: structure-invariant direct methods	Extinction coefficient: 0.0014 (2)

### Special details

**Table:**

Geometry. All e.s.d.'s (except the e.s.d. in the dihedral angle between two l.s. planes) are estimated using the full covariance matrix. The cell e.s.d.'s are taken into account individually in the estimation of e.s.d.'s in distances, angles and torsion angles; correlations between e.s.d.'s in cell parameters are only used when they are defined by crystal symmetry. An approximate (isotropic) treatment of cell e.s.d.'s is used for estimating e.s.d.'s involving l.s. planes.
Refinement. Refinement of *F*^2^ against ALL reflections. The weighted *R*-factor *wR* and goodness of fit *S* are based on *F*^2^, conventional *R*-factors *R* are based on *F*, with *F* set to zero for negative *F*^2^. The threshold expression of *F*^2^ > σ(*F*^2^) is used only for calculating *R*-factors(gt) *etc*. and is not relevant to the choice of reflections for refinement. *R*-factors based on *F*^2^ are statistically about twice as large as those based on *F*, and *R*- factors based on ALL data will be even larger.

### Fractional atomic coordinates and isotropic or equivalent isotropic displacement parameters (Å^2^)

**Table:**

	*x*	*y*	*z*	*U*_iso_*/*U*_eq_	
Hg1	0.24751 (3)	0.03490 (2)	0.406074 (17)	0.02741 (8)	
Hg2	0.28108 (3)	0.38606 (2)	0.552272 (16)	0.02422 (8)	
Br1	0.18713 (12)	0.04795 (8)	0.57243 (5)	0.04088 (18)	
Br2	0.55928 (8)	0.29653 (7)	0.48471 (5)	0.03010 (14)	
Fe1	0.45120 (10)	0.13645 (8)	0.16271 (6)	0.01728 (15)	
Fe2	0.01347 (10)	0.41228 (8)	0.76191 (6)	0.01701 (15)	
C1	0.2858 (7)	0.0275 (6)	0.2658 (4)	0.0228 (11)	
C2	0.1899 (7)	0.1127 (7)	0.1840 (5)	0.0250 (12)	
H2A	0.0979	0.1883	0.1845	0.030*	
C3	0.2543 (8)	0.0659 (7)	0.1011 (5)	0.0275 (12)	
H3A	0.2130	0.1046	0.0374	0.033*	
C4	0.3919 (8)	−0.0492 (7)	0.1313 (5)	0.0268 (12)	
H4A	0.4585	−0.1010	0.0912	0.032*	
C5	0.4121 (8)	−0.0729 (6)	0.2332 (5)	0.0237 (11)	
H5A	0.4948	−0.1430	0.2722	0.028*	
C6	0.5781 (8)	0.2740 (8)	0.2176 (5)	0.0305 (13)	
H6A	0.5635	0.2869	0.2808	0.037*	
C7	0.4793 (8)	0.3539 (6)	0.1362 (5)	0.0289 (13)	
H7A	0.3881	0.4305	0.1354	0.035*	
C8	0.5405 (8)	0.2994 (7)	0.0558 (5)	0.0277 (12)	
H8A	0.4969	0.3323	−0.0078	0.033*	
C9	0.6786 (8)	0.1869 (7)	0.0887 (5)	0.0269 (12)	
H9A	0.7443	0.1313	0.0505	0.032*	
C10	0.7021 (7)	0.1715 (7)	0.1882 (5)	0.0283 (13)	
H10A	0.7862	0.1042	0.2281	0.034*	
C11	0.0541 (7)	0.4524 (7)	0.6167 (4)	0.0223 (11)	
C12	−0.0904 (8)	0.3691 (7)	0.6451 (5)	0.0267 (12)	
H12A	−0.0992	0.2757	0.6353	0.032*	
C13	−0.2200 (8)	0.4497 (7)	0.6908 (5)	0.0282 (13)	
H13A	−0.3296	0.4195	0.7171	0.034*	
C14	−0.1548 (8)	0.5847 (7)	0.6898 (5)	0.0290 (13)	
H14A	−0.2138	0.6601	0.7152	0.035*	
C15	0.0141 (8)	0.5857 (6)	0.6441 (4)	0.0242 (11)	
H15A	0.0874	0.6620	0.6337	0.029*	
C16	0.1635 (10)	0.2313 (8)	0.8352 (5)	0.0332 (14)	
H16A	0.2081	0.1515	0.8101	0.040*	
C17	−0.0008 (10)	0.2464 (8)	0.8831 (5)	0.0357 (16)	
H17A	−0.0850	0.1781	0.8962	0.043*	
C18	−0.0171 (9)	0.3811 (9)	0.9082 (5)	0.0339 (14)	
H18A	−0.1149	0.4196	0.9403	0.041*	
C19	0.1387 (9)	0.4490 (8)	0.8768 (5)	0.0312 (13)	
H19A	0.1641	0.5399	0.8848	0.037*	
C20	0.2490 (8)	0.3556 (8)	0.8313 (5)	0.0315 (14)	
H20A	0.3614	0.3738	0.8031	0.038*	

### Atomic displacement parameters (Å^2^)

**Table:**

	*U*^11^	*U*^22^	*U*^33^	*U*^12^	*U*^13^	*U*^23^
Hg1	0.03585 (13)	0.02139 (12)	0.02352 (12)	−0.00544 (9)	0.00906 (9)	−0.00357 (9)
Hg2	0.02487 (12)	0.02667 (12)	0.01967 (12)	0.00328 (8)	0.00183 (8)	−0.00630 (9)
Br1	0.0666 (5)	0.0293 (3)	0.0270 (3)	−0.0080 (3)	0.0131 (3)	−0.0086 (3)
Br2	0.0271 (3)	0.0312 (3)	0.0310 (3)	0.0051 (2)	0.0038 (2)	−0.0104 (3)
Fe1	0.0159 (3)	0.0169 (3)	0.0182 (4)	−0.0034 (3)	0.0003 (3)	−0.0023 (3)
Fe2	0.0165 (3)	0.0165 (3)	0.0184 (4)	−0.0027 (3)	0.0009 (3)	−0.0048 (3)
C1	0.024 (2)	0.020 (2)	0.023 (3)	−0.010 (2)	0.008 (2)	−0.002 (2)
C2	0.017 (2)	0.025 (3)	0.033 (3)	−0.007 (2)	0.001 (2)	−0.005 (3)
C3	0.027 (3)	0.031 (3)	0.024 (3)	−0.013 (2)	−0.005 (2)	−0.002 (3)
C4	0.034 (3)	0.022 (3)	0.028 (3)	−0.008 (2)	0.000 (2)	−0.011 (2)
C5	0.027 (3)	0.019 (2)	0.024 (3)	−0.004 (2)	0.002 (2)	−0.002 (2)
C6	0.030 (3)	0.037 (3)	0.030 (3)	−0.016 (3)	0.003 (3)	−0.015 (3)
C7	0.027 (3)	0.016 (2)	0.042 (4)	−0.006 (2)	0.002 (3)	−0.003 (3)
C8	0.030 (3)	0.025 (3)	0.025 (3)	−0.013 (2)	0.003 (2)	0.003 (2)
C9	0.024 (3)	0.030 (3)	0.028 (3)	−0.007 (2)	0.010 (2)	−0.008 (3)
C10	0.016 (2)	0.033 (3)	0.033 (3)	−0.008 (2)	0.000 (2)	−0.002 (3)
C11	0.023 (2)	0.024 (3)	0.019 (3)	−0.001 (2)	0.001 (2)	−0.005 (2)
C12	0.026 (3)	0.032 (3)	0.026 (3)	−0.003 (2)	−0.007 (2)	−0.012 (3)
C13	0.020 (2)	0.032 (3)	0.034 (3)	−0.001 (2)	−0.005 (2)	−0.012 (3)
C14	0.025 (3)	0.025 (3)	0.033 (3)	0.005 (2)	0.006 (2)	−0.005 (3)
C15	0.025 (3)	0.020 (3)	0.025 (3)	0.002 (2)	0.007 (2)	−0.004 (2)
C16	0.042 (4)	0.027 (3)	0.027 (3)	0.007 (3)	−0.008 (3)	−0.004 (3)
C17	0.042 (4)	0.032 (3)	0.028 (3)	−0.018 (3)	−0.009 (3)	0.008 (3)
C18	0.028 (3)	0.049 (4)	0.025 (3)	−0.008 (3)	0.003 (3)	−0.011 (3)
C19	0.035 (3)	0.036 (3)	0.028 (3)	−0.011 (3)	−0.006 (3)	−0.013 (3)
C20	0.020 (3)	0.041 (4)	0.030 (3)	−0.002 (2)	−0.004 (2)	−0.003 (3)

### Geometric parameters (Å, º)

**Table:**

Hg1—C1	2.046 (6)	C3—C4	1.426 (9)
Hg1—Br1	2.4511 (9)	C3—H3A	0.9500
Hg1—Br2^i^	3.3558 (9)	C4—C5	1.438 (9)
Hg1—Br1^ii^	3.4895 (11)	C4—H4A	0.9500
Hg2—C11	2.045 (6)	C5—H5A	0.9500
Hg2—Br2	2.4562 (7)	C6—C10	1.415 (10)
Hg2—Br2^iii^	3.3142 (9)	C6—C7	1.416 (10)
Hg2—Br1	3.3341 (10)	C6—H6A	0.9500
Br1—Hg1^ii^	3.4895 (11)	C7—C8	1.428 (10)
Br2—Hg2^iii^	3.3142 (9)	C7—H7A	0.9500
Br2—Hg1^i^	3.3558 (9)	C8—C9	1.420 (9)
Fe1—C2	2.039 (5)	C8—H8A	0.9500
Fe1—C3	2.041 (6)	C9—C10	1.419 (9)
Fe1—C9	2.042 (6)	C9—H9A	0.9500
Fe1—C4	2.045 (6)	C10—H10A	0.9500
Fe1—C8	2.048 (6)	C11—C12	1.423 (8)
Fe1—C6	2.048 (6)	C11—C15	1.426 (8)
Fe1—C10	2.050 (6)	C12—C13	1.427 (9)
Fe1—C5	2.050 (6)	C12—H12A	0.9500
Fe1—C7	2.052 (6)	C13—C14	1.435 (9)
Fe1—C1	2.059 (5)	C13—H13A	0.9500
Fe2—C16	2.032 (7)	C14—C15	1.424 (8)
Fe2—C11	2.042 (6)	C14—H14A	0.9500
Fe2—C12	2.044 (6)	C15—H15A	0.9500
Fe2—C17	2.045 (7)	C16—C20	1.411 (10)
Fe2—C13	2.045 (6)	C16—C17	1.418 (11)
Fe2—C15	2.046 (6)	C16—H16A	0.9500
Fe2—C20	2.046 (6)	C17—C18	1.416 (11)
Fe2—C14	2.048 (6)	C17—H17A	0.9500
Fe2—C18	2.056 (7)	C18—C19	1.424 (9)
Fe2—C19	2.071 (6)	C18—H18A	0.9500
C1—C2	1.425 (9)	C19—C20	1.420 (10)
C1—C5	1.432 (8)	C19—H19A	0.9500
C2—C3	1.427 (9)	C20—H20A	0.9500
C2—H2A	0.9500		
			
C1—Hg1—Br1	177.32 (17)	C1—C2—C3	109.0 (5)
C1—Hg1—Br2^i^	99.46 (17)	C1—C2—Fe1	70.4 (3)
Br1—Hg1—Br2^i^	82.33 (3)	C3—C2—Fe1	69.6 (3)
C1—Hg1—Br1^ii^	96.81 (16)	C1—C2—H2A	125.5
Br1—Hg1—Br1^ii^	80.94 (3)	C3—C2—H2A	125.5
Br2^i^—Hg1—Br1^ii^	97.82 (2)	Fe1—C2—H2A	126.1
C11—Hg2—Br2	176.42 (17)	C4—C3—C2	107.7 (5)
C11—Hg2—Br2^iii^	94.10 (17)	C4—C3—Fe1	69.7 (3)
Br2—Hg2—Br2^iii^	87.16 (2)	C2—C3—Fe1	69.5 (3)
C11—Hg2—Br1	95.97 (17)	C4—C3—H3A	126.2
Br2—Hg2—Br1	82.77 (3)	C2—C3—H3A	126.2
Br2^iii^—Hg2—Br1	169.922 (19)	Fe1—C3—H3A	126.2
Hg1—Br1—Hg2	98.97 (3)	C3—C4—C5	107.9 (6)
Hg1—Br1—Hg1^ii^	99.06 (3)	C3—C4—Fe1	69.4 (3)
Hg2—Br1—Hg1^ii^	121.04 (3)	C5—C4—Fe1	69.6 (3)
Hg2—Br2—Hg2^iii^	92.84 (2)	C3—C4—H4A	126.0
Hg2—Br2—Hg1^i^	118.45 (3)	C5—C4—H4A	126.0
Hg2^iii^—Br2—Hg1^i^	128.28 (2)	Fe1—C4—H4A	126.5
C2—Fe1—C3	40.9 (3)	C1—C5—C4	108.1 (5)
C2—Fe1—C9	157.9 (3)	C1—C5—Fe1	69.9 (3)
C3—Fe1—C9	121.0 (3)	C4—C5—Fe1	69.3 (3)
C2—Fe1—C4	68.7 (3)	C1—C5—H5A	126.0
C3—Fe1—C4	40.8 (3)	C4—C5—H5A	126.0
C9—Fe1—C4	105.8 (3)	Fe1—C5—H5A	126.4
C2—Fe1—C8	122.1 (3)	C10—C6—C7	108.1 (6)
C3—Fe1—C8	104.7 (3)	C10—C6—Fe1	69.9 (4)
C9—Fe1—C8	40.6 (3)	C7—C6—Fe1	70.0 (3)
C4—Fe1—C8	119.7 (3)	C10—C6—H6A	125.9
C2—Fe1—C6	123.9 (3)	C7—C6—H6A	125.9
C3—Fe1—C6	158.2 (3)	Fe1—C6—H6A	125.8
C9—Fe1—C6	68.1 (3)	C6—C7—C8	108.3 (6)
C4—Fe1—C6	160.7 (3)	C6—C7—Fe1	69.6 (4)
C8—Fe1—C6	68.5 (3)	C8—C7—Fe1	69.5 (3)
C2—Fe1—C10	160.1 (3)	C6—C7—H7A	125.9
C3—Fe1—C10	158.3 (3)	C8—C7—H7A	125.9
C9—Fe1—C10	40.6 (3)	Fe1—C7—H7A	126.6
C4—Fe1—C10	123.2 (3)	C9—C8—C7	107.2 (6)
C8—Fe1—C10	68.4 (3)	C9—C8—Fe1	69.4 (3)
C6—Fe1—C10	40.4 (3)	C7—C8—Fe1	69.8 (3)
C2—Fe1—C5	68.5 (2)	C9—C8—H8A	126.4
C3—Fe1—C5	68.9 (3)	C7—C8—H8A	126.4
C9—Fe1—C5	122.2 (3)	Fe1—C8—H8A	125.9
C4—Fe1—C5	41.1 (3)	C10—C9—C8	108.5 (6)
C8—Fe1—C5	156.8 (3)	C10—C9—Fe1	70.0 (3)
C6—Fe1—C5	125.4 (3)	C8—C9—Fe1	69.9 (3)
C10—Fe1—C5	108.8 (3)	C10—C9—H9A	125.7
C2—Fe1—C7	108.0 (2)	C8—C9—H9A	125.7
C3—Fe1—C7	121.1 (3)	Fe1—C9—H9A	125.9
C9—Fe1—C7	68.1 (3)	C6—C10—C9	107.9 (6)
C4—Fe1—C7	156.2 (3)	C6—C10—Fe1	69.7 (3)
C8—Fe1—C7	40.7 (3)	C9—C10—Fe1	69.4 (3)
C6—Fe1—C7	40.4 (3)	C6—C10—H10A	126.0
C10—Fe1—C7	67.9 (3)	C9—C10—H10A	126.0
C5—Fe1—C7	161.5 (3)	Fe1—C10—H10A	126.4
C2—Fe1—C1	40.7 (2)	C12—C11—C15	107.9 (5)
C3—Fe1—C1	69.0 (3)	C12—C11—Fe2	69.7 (3)
C9—Fe1—C1	159.2 (3)	C15—C11—Fe2	69.7 (3)
C4—Fe1—C1	68.9 (3)	C12—C11—Hg2	125.1 (5)
C8—Fe1—C1	159.6 (3)	C15—C11—Hg2	126.9 (4)
C6—Fe1—C1	109.8 (3)	Fe2—C11—Hg2	124.6 (3)
C10—Fe1—C1	124.4 (3)	C11—C12—C13	108.4 (6)
C5—Fe1—C1	40.8 (2)	C11—C12—Fe2	69.6 (3)
C7—Fe1—C1	124.8 (3)	C13—C12—Fe2	69.6 (4)
C16—Fe2—C11	112.4 (3)	C11—C12—H12A	125.8
C16—Fe2—C12	108.9 (3)	C13—C12—H12A	125.8
C11—Fe2—C12	40.8 (2)	Fe2—C12—H12A	126.6
C16—Fe2—C17	40.7 (3)	C12—C13—C14	107.6 (6)
C11—Fe2—C17	142.1 (3)	C12—C13—Fe2	69.5 (3)
C12—Fe2—C17	112.4 (3)	C14—C13—Fe2	69.6 (4)
C16—Fe2—C13	134.6 (3)	C12—C13—H13A	126.2
C11—Fe2—C13	68.9 (3)	C14—C13—H13A	126.2
C12—Fe2—C13	40.8 (3)	Fe2—C13—H13A	126.3
C17—Fe2—C13	109.3 (3)	C15—C14—C13	107.9 (5)
C16—Fe2—C15	142.9 (3)	C15—C14—Fe2	69.6 (3)
C11—Fe2—C15	40.8 (2)	C13—C14—Fe2	69.4 (4)
C12—Fe2—C15	68.6 (3)	C15—C14—H14A	126.0
C17—Fe2—C15	176.2 (3)	C13—C14—H14A	126.0
C13—Fe2—C15	68.8 (3)	Fe2—C14—H14A	126.6
C16—Fe2—C20	40.5 (3)	C14—C15—C11	108.2 (5)
C11—Fe2—C20	110.1 (3)	C14—C15—Fe2	69.7 (4)
C12—Fe2—C20	135.1 (3)	C11—C15—Fe2	69.4 (3)
C17—Fe2—C20	68.1 (3)	C14—C15—H15A	125.9
C13—Fe2—C20	174.7 (3)	C11—C15—H15A	125.9
C15—Fe2—C20	114.0 (3)	Fe2—C15—H15A	126.5
C16—Fe2—C14	175.3 (3)	C20—C16—C17	108.1 (6)
C11—Fe2—C14	68.7 (2)	C20—C16—Fe2	70.3 (4)
C12—Fe2—C14	68.7 (3)	C17—C16—Fe2	70.1 (4)
C17—Fe2—C14	135.8 (3)	C20—C16—H16A	125.9
C13—Fe2—C14	41.0 (3)	C17—C16—H16A	125.9
C15—Fe2—C14	40.7 (2)	Fe2—C16—H16A	125.2
C20—Fe2—C14	144.0 (3)	C18—C17—C16	107.9 (6)
C16—Fe2—C18	68.2 (3)	C18—C17—Fe2	70.2 (4)
C11—Fe2—C18	176.5 (3)	C16—C17—Fe2	69.2 (4)
C12—Fe2—C18	142.7 (3)	C18—C17—H17A	126.0
C17—Fe2—C18	40.4 (3)	C16—C17—H17A	126.0
C13—Fe2—C18	113.4 (3)	Fe2—C17—H17A	126.1
C15—Fe2—C18	136.8 (3)	C17—C18—C19	108.1 (6)
C20—Fe2—C18	68.0 (3)	C17—C18—Fe2	69.4 (4)
C14—Fe2—C18	111.0 (3)	C19—C18—Fe2	70.4 (4)
C16—Fe2—C19	68.1 (3)	C17—C18—H18A	125.9
C11—Fe2—C19	136.3 (3)	C19—C18—H18A	125.9
C12—Fe2—C19	175.3 (3)	Fe2—C18—H18A	125.9
C17—Fe2—C19	68.0 (3)	C20—C19—C18	107.4 (6)
C13—Fe2—C19	143.8 (3)	C20—C19—Fe2	68.9 (4)
C15—Fe2—C19	111.4 (3)	C18—C19—Fe2	69.2 (4)
C20—Fe2—C19	40.3 (3)	C20—C19—H19A	126.3
C14—Fe2—C19	114.6 (3)	C18—C19—H19A	126.3
C18—Fe2—C19	40.4 (3)	Fe2—C19—H19A	127.1
C2—C1—C5	107.3 (5)	C16—C20—C19	108.4 (6)
C2—C1—Hg1	127.8 (4)	C16—C20—Fe2	69.2 (4)
C5—C1—Hg1	124.8 (5)	C19—C20—Fe2	70.8 (4)
C2—C1—Fe1	68.9 (3)	C16—C20—H20A	125.8
C5—C1—Fe1	69.3 (3)	C19—C20—H20A	125.8
Hg1—C1—Fe1	129.4 (3)	Fe2—C20—H20A	125.8
			
C5—C1—C2—C3	0.3 (6)	C15—C11—C12—C13	−0.4 (7)
Hg1—C1—C2—C3	−176.7 (4)	Fe2—C11—C12—C13	59.0 (4)
Fe1—C1—C2—C3	59.1 (4)	Hg2—C11—C12—C13	177.7 (4)
C5—C1—C2—Fe1	−58.8 (4)	C15—C11—C12—Fe2	−59.4 (4)
Hg1—C1—C2—Fe1	124.2 (4)	Hg2—C11—C12—Fe2	118.7 (4)
C1—C2—C3—C4	−0.1 (6)	C11—C12—C13—C14	0.4 (7)
Fe1—C2—C3—C4	59.5 (4)	Fe2—C12—C13—C14	59.4 (5)
C1—C2—C3—Fe1	−59.6 (4)	C11—C12—C13—Fe2	−59.0 (4)
C2—C3—C4—C5	−0.1 (6)	C12—C13—C14—C15	−0.3 (8)
Fe1—C3—C4—C5	59.2 (4)	Fe2—C13—C14—C15	59.1 (5)
C2—C3—C4—Fe1	−59.3 (4)	C12—C13—C14—Fe2	−59.4 (4)
C2—C1—C5—C4	−0.4 (6)	C13—C14—C15—C11	0.0 (7)
Hg1—C1—C5—C4	176.7 (4)	Fe2—C14—C15—C11	59.0 (4)
Fe1—C1—C5—C4	−58.9 (4)	C13—C14—C15—Fe2	−59.0 (5)
C2—C1—C5—Fe1	58.6 (4)	C12—C11—C15—C14	0.3 (7)
Hg1—C1—C5—Fe1	−124.3 (4)	Fe2—C11—C15—C14	−59.2 (4)
C3—C4—C5—C1	0.3 (7)	Hg2—C11—C15—C14	−177.8 (5)
Fe1—C4—C5—C1	59.4 (4)	C12—C11—C15—Fe2	59.4 (4)
C3—C4—C5—Fe1	−59.1 (4)	Hg2—C11—C15—Fe2	−118.6 (5)
C10—C6—C7—C8	0.8 (7)	C20—C16—C17—C18	0.5 (8)
Fe1—C6—C7—C8	−58.9 (4)	Fe2—C16—C17—C18	−59.8 (5)
C10—C6—C7—Fe1	59.7 (4)	C20—C16—C17—Fe2	60.3 (5)
C6—C7—C8—C9	−0.7 (6)	C16—C17—C18—C19	−0.8 (8)
Fe1—C7—C8—C9	−59.6 (4)	Fe2—C17—C18—C19	−59.9 (5)
C6—C7—C8—Fe1	59.0 (4)	C16—C17—C18—Fe2	59.1 (5)
C7—C8—C9—C10	0.3 (7)	C17—C18—C19—C20	0.8 (8)
Fe1—C8—C9—C10	−59.6 (4)	Fe2—C18—C19—C20	−58.5 (5)
C7—C8—C9—Fe1	59.9 (4)	C17—C18—C19—Fe2	59.3 (5)
C7—C6—C10—C9	−0.7 (7)	C17—C16—C20—C19	0.0 (8)
Fe1—C6—C10—C9	59.1 (4)	Fe2—C16—C20—C19	60.2 (5)
C7—C6—C10—Fe1	−59.7 (4)	C17—C16—C20—Fe2	−60.2 (5)
C8—C9—C10—C6	0.2 (7)	C18—C19—C20—C16	−0.5 (8)
Fe1—C9—C10—C6	−59.3 (4)	Fe2—C19—C20—C16	−59.2 (5)
C8—C9—C10—Fe1	59.5 (4)	C18—C19—C20—Fe2	58.7 (5)

Symmetry codes: (i) −*x*+1, −*y*, −*z*+1; (ii) −*x*, −*y*, −*z*+1; (iii) −*x*+1, −*y*+1, −*z*+1.

### Hydrogen-bond geometry (Å, º)

Cg1 and Cg2 are the centroids of the C6–C10 and C16–C20 rings, respectively.

**Table:**

*D*—H···*A*	*D*—H	H···*A*	*D*···*A*	*D*—H···*A*
C3—H3*A*···*Cg*2^iv^	0.95	2.91	3.794 (8)	156
C19—H19*A*···*Cg*1^iii^	0.95	2.78	3.618 (8)	148

Symmetry codes: (iii) −*x*+1, −*y*+1, −*z*+1; (iv) *x*, *y*, *z*−1.
